# Chemical Constituents from the Stems of *Manihot esculenta*

**DOI:** 10.1007/s13659-015-0052-8

**Published:** 2015-02-12

**Authors:** Ya-Mei Pan, Yu Zhang, Xiao-Nan Wang, He-Ping Chen, Shun-Lin Li, Ying-Tong Di, Duo-Zhi Chen, Ling-Li Guo, Xiao-Jiang Hao, Hong-Ping He

**Affiliations:** 1 State Key Laboratory of Phytochemistry and Plant Resources in West China, Kunming Institute of Botany, Chinese Academy of Sciences, Kunming, 650201 Yunnan People’s Republic of China; 2 University of Chinese Academy of Sciences, Beijing, 100039 People’s Republic of China; 3 School of Pharmacy, Yunnan University of TCM, Kunming, 650500 Yunnan People’s Republic of China

**Keywords:** Euphorbiaceae, *Manihot esculenta*, Chemical constituents, Diterpenoids

## Abstract

**Abstract:**

Two new compounds, maniesculentins A (**1**) and B (**6**), together with four known ones were isolated from the stems of *Manihot esculenta* Crantz. The structures of the new compounds were elucidated by extensive spectroscopic methods including NMR spectroscopy and mass spectrometry. The two new compounds (**1**, **6**) were assayed for antibacterial activity against four tested bacteria lines.

**Graphical Abstract:**

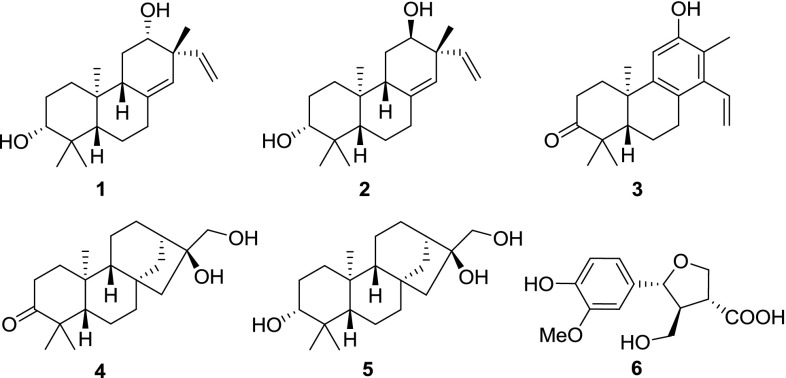

**Electronic supplementary material:**

The online version of this article (doi:10.1007/s13659-015-0052-8) contains supplementary material, which is available to authorized users.

## Introduction

**Fig. 1:**
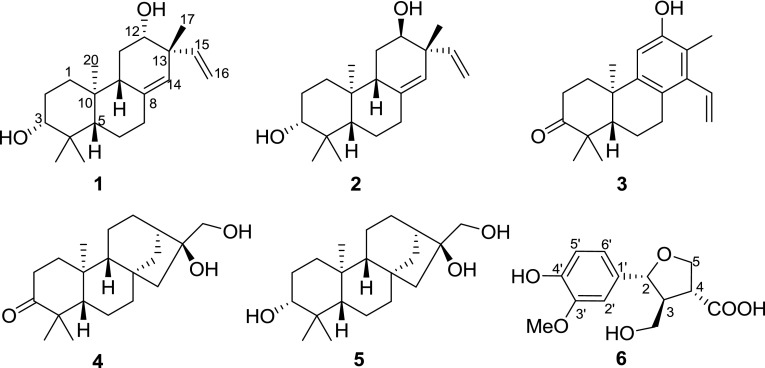
Chemical structures of compounds **1**–**6**

The Euphorbiaceae produce a diverse range of secondary metabolites. Recently, a series of secondary metabolites with fascinating structural features and significant biological activities from Euphorbiaceae family were reported. The main constitutes of Euphorbiaceae are diterpenoids with different skeletons such as jatrophanes, lathyranes, tiglianes, ingenanes, and myrsinanes with a wide array of pharmaceutical activities, such as antiproliferative, cytotoxic, antimicrobial and anti-inflammatory, anticancer and antioxidant activities [1–7]. *Manihot* (Euphorbiaceae), a shrub, has about 60 species and is widely cultivated in tropical regions. Two species were introduced in China, which are *M. esculenta* Crantz and *M.* *glaziovii*. *M. esculenta* is widely cultivated in Fujian, Guangdong, Guangxi, Guizhou, Hainan, Taiwan and Yunnan provences of China [8]. Previous study on *M. esculenta* roots reported various types of stress metabolites, which are predominantly steroids and diterpenoids produced in the damaged cassava root tissue by cutting and fungal-infection [9]. Recently investigations in China mainly focused on the roots distributed in Hainan [10]. However, the study on the plant distributed in Yunnan has not yet reported. Therefore, searching for novel structural and bioactive natural products from its stems led to the isolation of two new compounds **1** and **6** (Fig. 1), and four known diterpenoids, yucalexin P**–**21 (**2**) [10, 11], cleistanthene**–**type sonderianol (**3**) [12], calliterpenone (**4**) [13], *ent*-kauran-3*α*,16*α*, 17-triol (**5**) [14]. Herein, this paper describes the isolation, structure elucidation and antimicrobial activities of these compounds.

## Results and Discussion

**Fig. 2:**
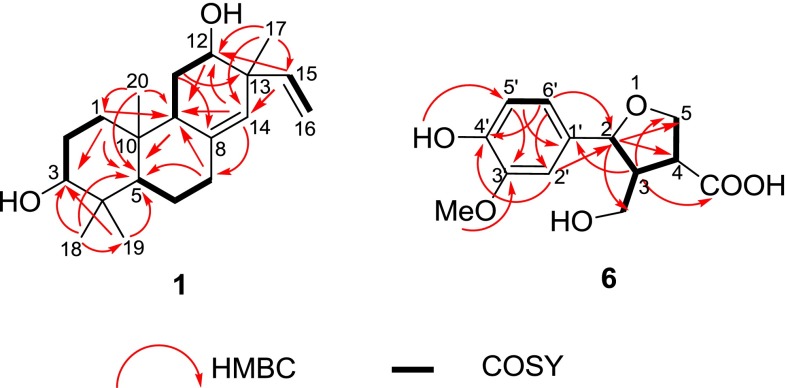
Key ^1^H-^1^H COSY and HMBC correlations of **1** and **6**

**Fig. 3:**
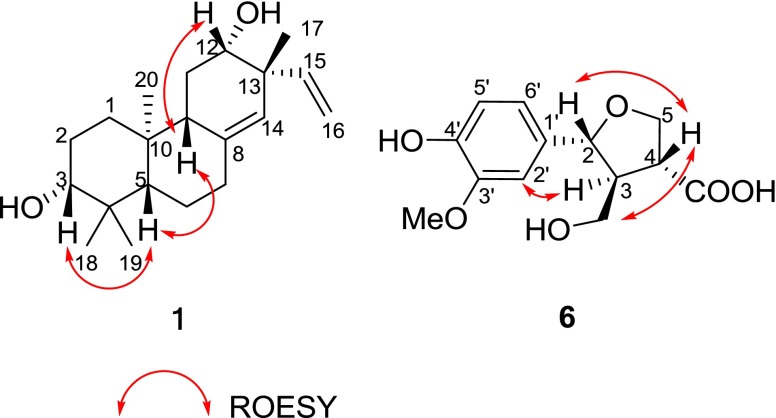
Key ROESY correlations of **1** and **6**

Maniesculentin A (**1**) was isolated as a white, amorphous powder. Its molecular formula C_20_H_32_O_2_ was determined by HREIMS ([M]^+^ *m/z* 304.2408, calcd. 304.2402), indicating five degrees of unsaturation. The IR absorption signals revealed the presence of hydroxyl (3430 cm^−1^) and olefinic (1634 cm^−1^) groups. The ^1^H NMR spectrum (Table 1) of **1** exhibited four angular methyl group (*δ*_H_ 0.74, 0.82, 1.01 and 1.07), two oxygenated methines [*δ*_H_ 3.26 (1H, dd, J = 11.4 and 4.1 Hz), 3.63 (1H, s); *δ*_*C*_ 78.8, 72.7], one trisubstituted double bond [*δ*_H_ (5.07, s); *δ*_*C*_ 138.1, 125.7], and one terminal double bond [*δ*_*H*_ 5.01(1H, d, *J* = 17.6 Hz), 5.00 (1H, d, *J* = 10.0 Hz), 5.74 (1H, dd, *J* = 17.6, 10.0 Hz); *δ*_*C*_ 146.1, 114.2], which were supported by HSQC and HMBC experiments (Fig. 2). The above information suggested that compound **1** should be an *ent*-pimarane-type diterpenoid. Detailed analysis of NMR data indicated that **1** was an isomer of **2** [10, 11], as indicated by the significant variation of ^13^C NMR signals at *δ*_*C*_ 25.9 for C-11, 43.7 for C-13 and 23.5 for Me-17 in **1** instead of *δ*_*C*_ 36.8 for C-11, 47.5 for C-13 and 26.0 for Me-17 in **2**, suggesting the *β*–orientation for 12-OH. In addition, the ROESY correlation (Fig. 3) of H-9/H-12 further indicated *β*–orientation for 12-OH. The above elucidation was further confirmed by 2D NMR (HSQC, ^1^H-^1^H COSY, and HMBC) (Fig. 2). Firstly, the ^1^H-^1^H COSY (Fig. 2) revealed the presence of partial structures of –CH_2_-CH_2_-CH (O)-, -CH-CH_2_-CH_2_- and –CH_2_-CH (O)-, as shown with bold lines in Fig. 2. Secondly, the HBMC correlations (Fig. 2) of both two methyl prontons H_3_-19 (*δ*_*H*_ 0.82) and H_3_-18 (*δ*_*H*_ 1.02) with C-3 (*δ*_*C*_)/C-5 (*δ*_*C*_ 54.1)/C-4 (*δ*_*C*_ 38.9); the other two methyl prontons H_3_-20 (*δ*_*H*_ 0.74), and H_3_-17 (*δ*_*H*_ 1.07) with C-1 (*δ*_*C*_ 36.7)/C-5 (*δ*_*C*_ 54.1)/C-9 (*δ*_*C*_ 46.3)/C-10 (*δ*_*C*_ 38.2), and C-12 (*δ*_*C*_ 72.7)/C-13 (*δ*_*C*_ 43.7)/C-14 (*δ*_*C*_ 125.7), respectively; the H-7 (*δ*_*H*_ 2.37 and 3.10) with C-5 (*δ*_*C*_ 54.1)/C-10 (*δ*_*C*_ 38.2)/C-14 (*δ*_*C*_ 125.7); and the H-11 (*δ*_*H*_ 1.65) with C-8 (*δ*_*C*_ 138.1)/C-13 (*δ*_*C*_ 43.7) constructed the typical fused A/B/C- ring system of the *ent*-pimarane-type diterpenoid core. The relative configuration of 3-OH was *a*–orientation determined by the ROESY correlation (Fig. 3) from H-3 to H-5 and the large coupling constant (dd, *J* = 11.4 and 4.1 Hz) of H-3 with H_2_-2 [15]. Thus, the structure of **1** was determined as 3*a*,12*a*-dihydroxy-pimara-8(14),15-dien.

**Table 1:** NMR Data of **1** and **6** in CDCl_3_ (*δ* in ppm and *J* in Hz)

No.	**1**		No.	**6**	
	*δ*_H_	*δ*_C_		*δ*_H_	*δ*_C_
1	1.65 (m)	36.7	2	4.62 (d, 7. 0)	86.1
	1.16 (td, 13.4, 3.6)				
2	1.64 (m)	27.4	3	3.13 (m)	48.2
	1.54 (m)				
3	3.26 (dd, 11.4, 4.1)	78.8	4	3.46 (td, 9.0, 3.9)	46.0
4		38.9	5	4.37 (d, 9.0)	69.8
				4.19 (dd, 9.0, 3.9)	
5	1.09 (m)	54.1	1′		130.5
6	1.67 (m)	22.2	2′	6.89 (br s)	108.4
	1.43 (dd, 13.0, 4.1)				
7	2.37 (dd, 13.9, 5.0)	35.4	3′		146.9
	2.10 (td, 13.9, 5.0)				
8		138.1	4′		145.8
9	1.83 (t, 8.3)	46.3	5′	6.91 (d, 8.1)	114.4
10		38.2	6′	6.81 (dd, 8.1, 1.9)	119.1
11	1.68 (m)	25.9	CH_2_OH	4.51 (dd, 9.8, 7.0)	69.9
	1.62 (m)			4.33 (dd, 9.8, 1.9)	
12	3.63 (s)	72.7	COOH		178.6
13		43.7	OCH_3_	3.92 (s)	55.9
14	5.07 (s)	125.7			
15	5.74 (dd, 17.6, 10.0)	146.1			
16	5.01 (d, 17.6)	114.2			
	5.00 (d, 10.0)				
17	1.07 (s)	23.5			
18	1.02 (s)	28.4			
19	0.82 (s)	15.6			
20	0.74 (s)	14.7

Maniesculentin B (**6**) was obtained as colorless oil. Its molecular formula was deduced to be C_13_H_16_O_6_ from the HREIMS at *m/z* 268.0943 (calcd. 268.0947), corresponding to six degrees of unsaturation. The IR spectrum showed absorption bands at 3442, 1764, 1631 and 1517 cm^−1^, indicating the presence of hydroxyl, carbonyl and aromatic groups, respectively. The ^13^C NMR and DEPT spectroscopic data (Table 1) exhibited 13 carbon signals, including one 1,3,4-trisubstituted aromatic ring (*δ*_*C*_ 146.9, 145.8, 130.5, 19.1, 114.4, 108.4), one furan ring (*δ*_*C*_ 86.1, 46.0, 48.2, 69.9), one hydroxymethyl (*δ*_*C*_ 69.8), and one methoxy group (*δ*_*C*_ 55.9). The ^1^H NMR spectrum also revealed characteristic signals of 1, 3, 4-trisubstituted aromatic ring [*δ*_H_ 6.89 (br s, H-2), 6.91 (d, *J* = 8.1 Hz, H-5), 6.81 (br d, *J* = 8.1 Hz, H-6)]. The ^1^H NMR and ^13^C NMR spectra of **6** displayed similarity to the known lariciresinol, a furan methanol lignan isolated from *Araucaria angustifolia* [16]. The major difference is one carbonxyl group instead of 4′-hydroxy-3′-methoxyphenmethyl group at C-4 supported by the HMBC correlation (Fig. 2) from H-4 (*δ*_*H*_ 3.46) to COOH (*δ*_C_ 178.6). The hydroxymethy group was positioned at C-3 by means of the ^1^H–^1^H COSY correlation of H-3 (*δ*_H_ 3.13) with the two protons of CH_2_OH (*δ*_H_ 4.33 and 4.51), and the HMBC (Fig. 2) correlation from H-2 (*δ*_H_ 4.62) to CH_2_OH (*δ*_C_ 69.9). Addition, in the HMBC spectrum (Fig. 2), the correlations from OH (*δ*_H_ 5.63) to C-5′ (*δ*_C_ 114.4) and OMe (*δ*_C_ 55.9) to C-3′ (*δ*_C_ 146.9) indicated that OH and OMe were connected to C-4′ and C-3, respectively. On the basis of the above information, the planar structure of **6** was further confirmed in Fig. 1.

The ROESY correlation (Fig. 3) between H-2 and H-4 manifested that H-2 and H-4 were in the same side (assigned as *β*-orientation). The relative configuration of H-3 was *a*-orientation determined by coupling constants (*J* = 7.0 Hz), which was similar to the literature [17]. In addition, the ROESY correlations (Fig. 3) of CH_2_OH/H-4 and H-3/H-2′ further confirmed the relative configuration of H-3. Consequently, compound **6** was determined to be tetrahydro-2*a*-(4′-hydroxy-3′-methoxyphenyl)- 4*a*-carbonxyl-3*b*- hydroxymethyl furan (**6**), and named as maniesculentin B.

Maniesculentins A (**1**) and B (**6**) were assayed for antibacterial activicity against four bacteria lines (*Staphylococcus aureus*, *Streptococcus*, *Escherichia.coli*, *Pseudomonas aeruginosa*) by double**–**dilution [18, 19]. The results of antibacterial activity showed that the two new compounds were inactive against four tested bacteria lines.

## Experimental Part

### General Experimental Procedures

Optical rotations were detected with a JASCO P-1020 digital polarimeter. UV spectra were recorded on a Shimadzu UV**–**2401 PC spectrophotometer. IR spectra were scanned with Bruker Tensor**–**27 infrared spectrometer with a KBr disk. 1D and 2D NMR spectra were measured on Bruker AM**–**400, DRX**–**600 spectrometer using TMS as internal standard. MS and HREIMS spectra were carried out on Brucker HCT/E squire and Waters Autospec Premier P776 spectrum. HPLC analysis was performed on an Agilent 1100 liquid chromatograph equipped with a Waters X-Bridge C18 column (4.6 × 250 mm, 5 μm) with a flow rate of 3.0 *m*L/min, detected by a DAD detector. Column chromatography was carried out on silica gel (200–300 and 300–400 mesh; Qingdao Marine Chemical, Inc., Qingdao, P. R. China) and Sephadex LH**–**20 (40–70 μm, Amersham Pharmacia Biotech AB, Uppsala, Sweden). TLC spots were visualized under UV light and by dipping into 8 % H_2_SO4 in EtOH followed by heating.

### Plant Material

The stems of *M. esculenta* Crantz were collected from Xishuangbanna, Yunnan Province, People’s Republic of China, in June 2012. The plant was authenticated by Mr. Yu Chen (Kunming Institute of Botany, Chinese Academy of Sciences). A voucher specimen (No. H201206162) was deposited at the Key Laboratory of Phytochemistry and Plant Resources in West China, Kunming Institute of Botany, Chinese Academy of Sciences.

### Extraction and Isolation

The air-dried and powdered stems of *M. esculenta* (13.0 kg) were extracted with 95 % aqueous EtOH (3 × 20 L) under reflux for three times (4, 3, and 3 h, respectively). The combined EtOH extracts were concentrated under vacuum to give a crude extract (750.0 g), which was suspended in water and then partitioned with EtOAc. The EtOAc extract (140.0 g) was subjected to a silica gel column, eluted with petroleum ether-ethyl acetate (from 9:1 to 5:5) and then eluted with chloroform–methanol (from 9:1 to 8:2) to yield seven fractions (A–J). Fr. C (30.0 g) was separated over an MCI–gel column (MeOH/H_2_O from 4:6 to 10:0) to obtain four fractions (Fr. C1–C4). Fr. C3 (800.0 mg) was chromatographed on Sephadex LH–20 (MeOH) to obtain Fr. C3A (400.0 mg), which was further purified by a silica gel column (petroleum ether–acetone, 9:1) to obtain Fr. C3A2 (200.0 mg).

Fr. C3A2 was further purified by Semi**–**preparative HPLC using a Waters X-bridge C18 (4.6 × 250 mm, 5 *μ*m) column with 40 % MeOH/H_2_O to obtain compound **1** (10.0 mg) and **2** (16.0 mg). Fr.C2 (600.0 mg) was purified using Sephadex LH-20 (CHCl_3_–MeOH, 1:1) and then by the Waters X-bridge C18 (4.6 × 250 mm, 5 *μ*m) column with 60 % MeOH/H_2_O to afford compounds **3** (8.0 mg), **4** (5.0 mg), **5** (20.0 mg). Fr.C1 (102.0 mg) was chromatographed on Sephadex LH**–**20 (MeOH) to obtain Fr. C1A (56.0 mg), and further purified by a silica gel column (CHCl_3_) to obtain compound **6** (9.0 mg).

### Maniesculentins (**1**)

Amorphous powder. [*a*]_*D*_^23^ = −51. 7 (*c* = 0.40, CHCl_3_), UV (MeOH) *λ*_max_ (log *ε*) 204 (2.08); IR (KBr) *ν*_max_ 3443, 1633, 1456, 1385, 1179, 1038, 596 cm^−1^; ^1^H NMR and ^13^C NMR data, see (Table 1); positive ESIMS *m/z* 305 [M + H]^+^; HREIMS *m/z* 304.2408 [M]^+^ (calcd for C_13_H_16_O_6_, 304.2402).

### Maniesculentins B (**6**)

Colorless oil. [*a*]_*D*_^23^ = + 14. 7 (*c* = 0.60, CHCl_3_), UV (CHCl_3_) *λ*_max_ (log *ε*) 280 (2.39), 240 (2.33); IR (KBr) *ν*_max_ 3442, 1764, 1631, 1517, 1384, 1277, 1038, 575 cm^−1^; ^1^H NMR and ^13^C NMR data, see (Table 1); negative ESIMS *m/z* 267 [M-H]−; HREIMS *m/z* 268.0943 [M]^+^ (calcd for C_13_H_16_O_6_, 268.0943).

## Electronic supplementary material

Below is the link to the electronic supplementary material.

Supplementary material 1 (DOC 2575 kb)

## References

[1] Gao S, Liu HY, Wang YH, He HP, Wang JS, Di YT, Li CS, Fang X, Hao XJ (2007). Org. Lett..

[2] Tang GH, Zhang Yu, Yuan CM, Li Y, Gu YC, Di YT, Wang YH, Zuo GY, Li SF, Li SL, He HP, Hao XJ (2012). J. Nat. Prod..

[3] Li SF, Di YT, Li SL, Zhang Y, Yang FM, Sun QY, Simo JM, He HP, Hao XJ (2011). J. Nat. Prod..

[4] Özbilgin S, Citoğlu GS (2012). Turk J. Pharm. Sci..

[5] Bittner M, Alarcon J, Aqueveque P, Becerra J, Hernandez V, Hoeneisen M, Silva YM (2001). Bol. Soc. Chil. Quim..

[6] Wang Q, Zhang DY, Wu XM (2009). J. Pharm. Univ..

[7] Liu JQ, Yang YF, Li XY, Liu EQ, Li ZR, Zhou L, Li Y, Qiu MH (2013). Phytochemistry.

[8] Editorial Committee Flora of China (2008). Chinese Academy of Sciences.

[9] Sakai T, Nakagawa Y, Uritani I, Data ES (1986). Agric. Biol. Chem..

[10] Li SS, Dai HF, Zhao YX, Zuo WJ, Li XN, Mei WL (2012). J. Trop. Subtrop. Bot..

[11] Tsutomu S, Yoshiko N (1988). Phytochemistry.

[12] Grace MH, Jin YH, Wilson GR, Coates RM (2006). Phytochemistry.

[13] Liu G, Mvller R, Rvedi P (2003). Helv. Chim. Acta.

[14] Dou DQ, Tian F, Qiu YK, Xiang Z, Xu BX, Kang TG, Dong F (2010). Nat. Prod. Res..

[15] Denton RW, Harding WW, Anderson CI, Jacobs H, McLean S, Reynolds WF (2001). J. Nat. Prod..

[16] Fonseca SF, Cambello PI, Barata LES, Rubeda EA (1978). Phytochemistry.

[17] Junei K, Hiroyuki H, Katsura F, Toshihiro N (1991). Chem. Pharm. Bull..

[18] Zuo GY, Meng FY, Hao XY, Zhang YL, Wang GC, Xu GL (2008). J. Pharm. Pharm. Sci..

[19] Tang GH, Zhang Y, Gu YC, Li SF, Di YT, Wang YH, Yang CX, Zuo GY, Li SL, He HP, Hao XJ (2012). J. Nat. Prod..

