# Supplementary material for: Chemical Constituents from the Stems of Manihot esculenta
Source: Nat Prod Bioprospect. 2015 Feb 12;5(1):55–9. doi: 10.1007/s13659-015-0052-8 (PMC4328001; doi:10.1007/s13659-015-0052-8)
Supplement: Supplementary file 1 — Supplementary material 1 (DOC 2575 kb) [file 13659_2015_52_MOESM1_ESM.doc]

**Supplementary Material**

Chemical Constituents from the Stems of *Manihot esculenta*

Ya-Mei Pan,^a,b^ Yu Zhang,^a^ Xiao-Nan WANG,^c^ He-Ping Chen,^a,b^ Shun-Lin Li,^a^ • Ying-Tong Di,^a^ Duo-Zhi Chen,^a^ Ling-Li Guo,^a^ Xiao-Jiang Hao,^a*^ Hong-Ping He ^a,c*^

^a^State Key Laboratory of Phytochemistry and Plant Resources in West China,

Kunming Institute of Botany, Chinese Academy of Sciences, Kunming 650201, Yunnan, P. R. China

^b^University of Chinese Academy of Sciences, Beijing 100039, P. R. China

^c^School of Pharmacy,Yunnan University of TCM, Kunming 650500, Yunnan, P. R. China

To whom correspondence should be addressed. E-mail: [hehongping@mail.kib.ac.cn](mailto:hehongping@mail.kib.ac.cn), or [hehongping@yahoo.com](mailto:hehongping@yahoo.com) (H.P. He); [haoxj@mail.kib.ac.cn](mailto:haoxj@mail.kib.ac.cn) (X.J. Hao)

Maniesculentin A (**1**)

**Figure S1.** ^1^H NMR spectrum of compound **1** (CDCl_3_, 400 MHz)

**Figure S2.** ^13^C NMR and DEPT spectra of compound **1** (CDCl_3_, 100 MHz).

**Figure S3.** ^1^H-^1^H COSY spectrum of compound **1** (CDCl_3_).

**Figure S4.** HSQC spectrum of compound **1** (CDCl_3_).

**Figure S5.** HMBC spectrum of compound **1** (CDCl_3_).

**Figure S6.** ROESY spectrum of compound **1** (CDCl_3_)

**Figure S7.** IR spectrum of compound **1** (KBr disks).

**Figure S8.** UV spectrum of compound **1** (MeOH)

**Figure S9.** HREIMS of compound **1**

Maniesculentin B (**6**)

**Figure S10.** ^1^H NMR spectrum of compound **6** (CDCl_3_, 400 MHz).

**Figure S11.** ^13^C NMR and DEPT spectra of compound **6** (CDCl_3_, 100 MHz).

**Figure S12.** ^1^H-^1^H COSY spectrum of compound **6** (CDCl_3_).

**Figure S13.** HSQC spectrum of compound **6** (CDCl_3_).

**Figure S14.** HMBC spectrum of compound **6** (CDCl_3_).

.**Figure S15.** ROESY spectrum of compound **6** (CDCl_3_)

**Figure S16.** IR spectrum of compound **6** (KBr disks).

**Figure S17.** UV spectrum of compound **6** (MeOH)

**Figure S18.** HREIMS of compound **6**.


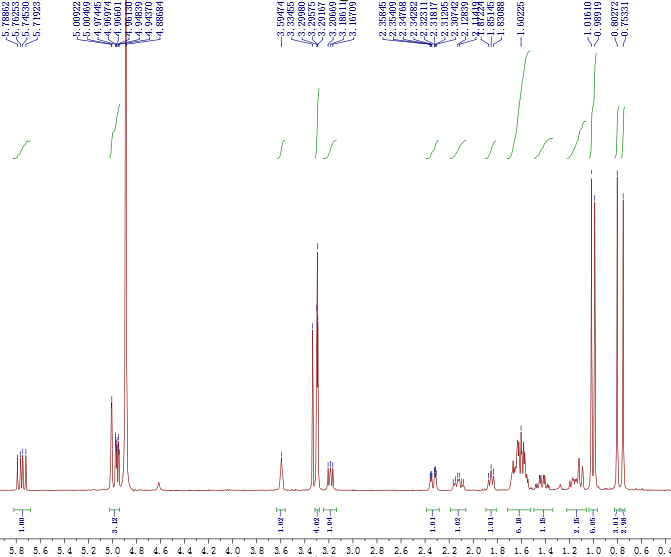


**Figure S1.** ^1^H NMR spectrum of compound **1** (CDCl_3_, 400 MHz)


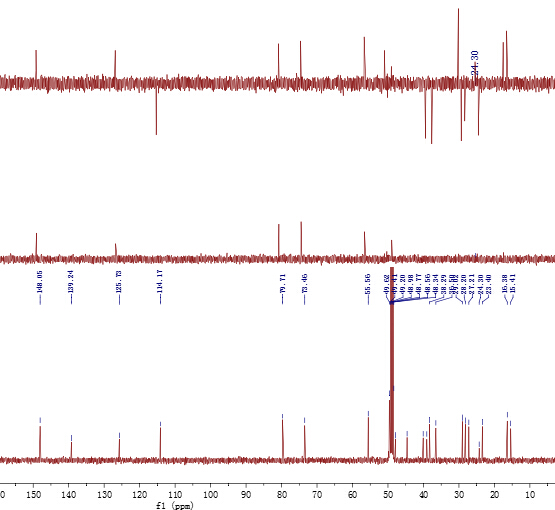


**Figure S2.** ^13^C NMR and DEPT spectrum of compound **1** (CDCl_3_, 100 MHz)


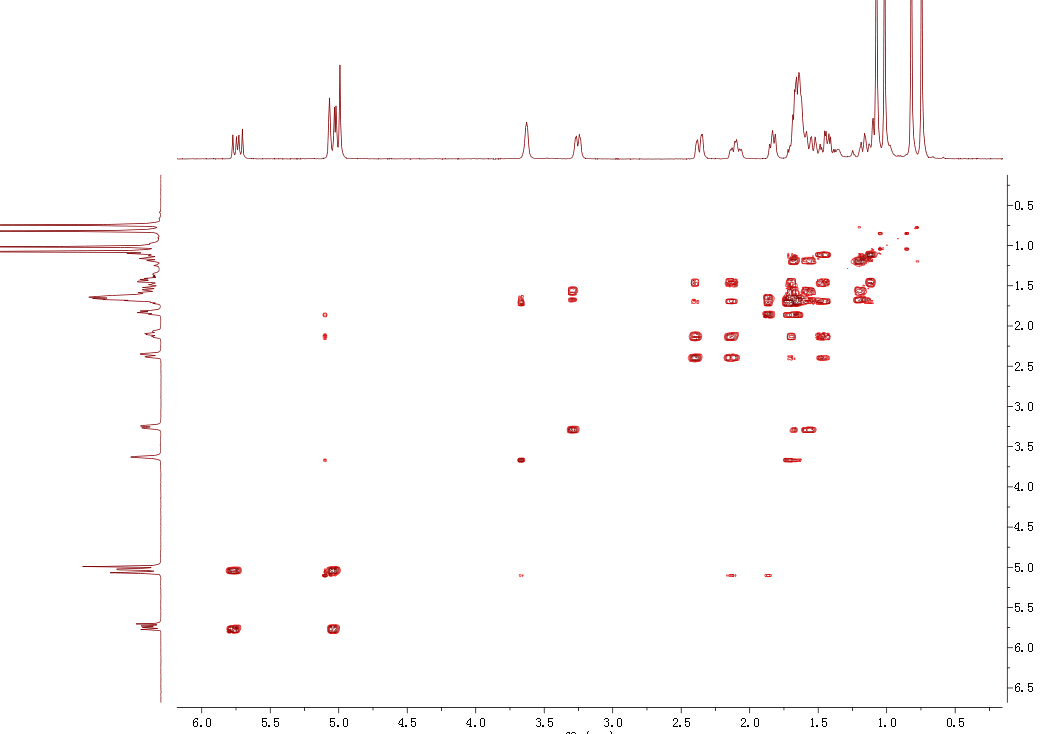


**Figure S3.** ^1^H-^1^H COSY spectrum of compound **1** (CDCl_3_)


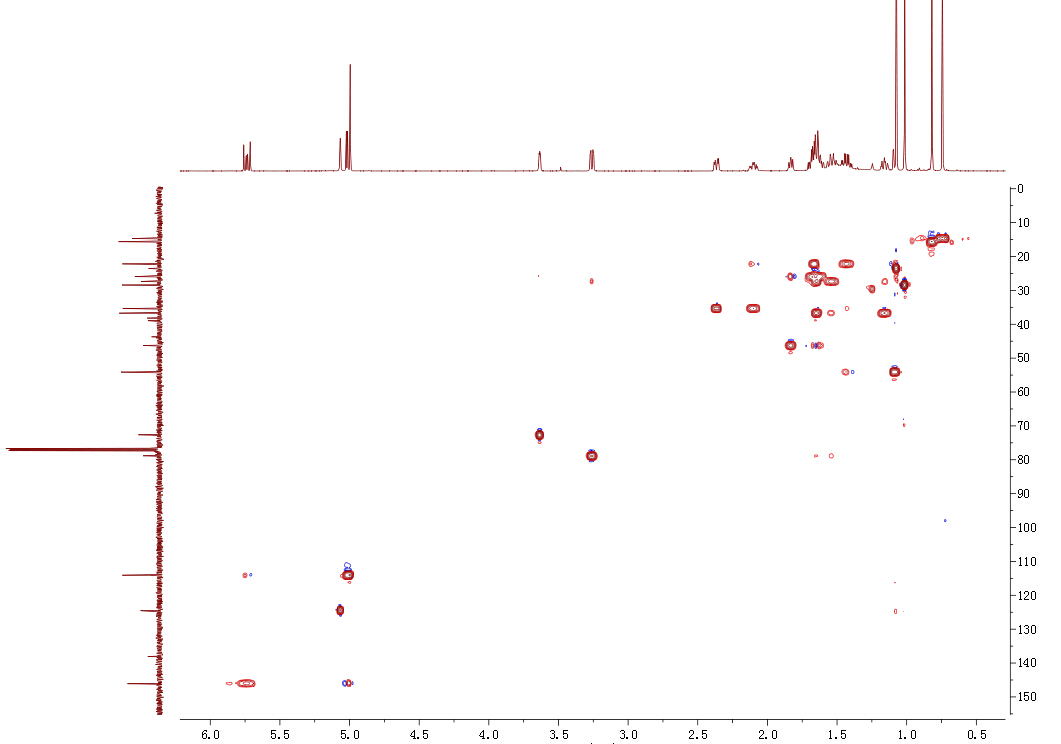


**Figure S4.** HSQC spectrum of compound **1** (CDCl_3_)


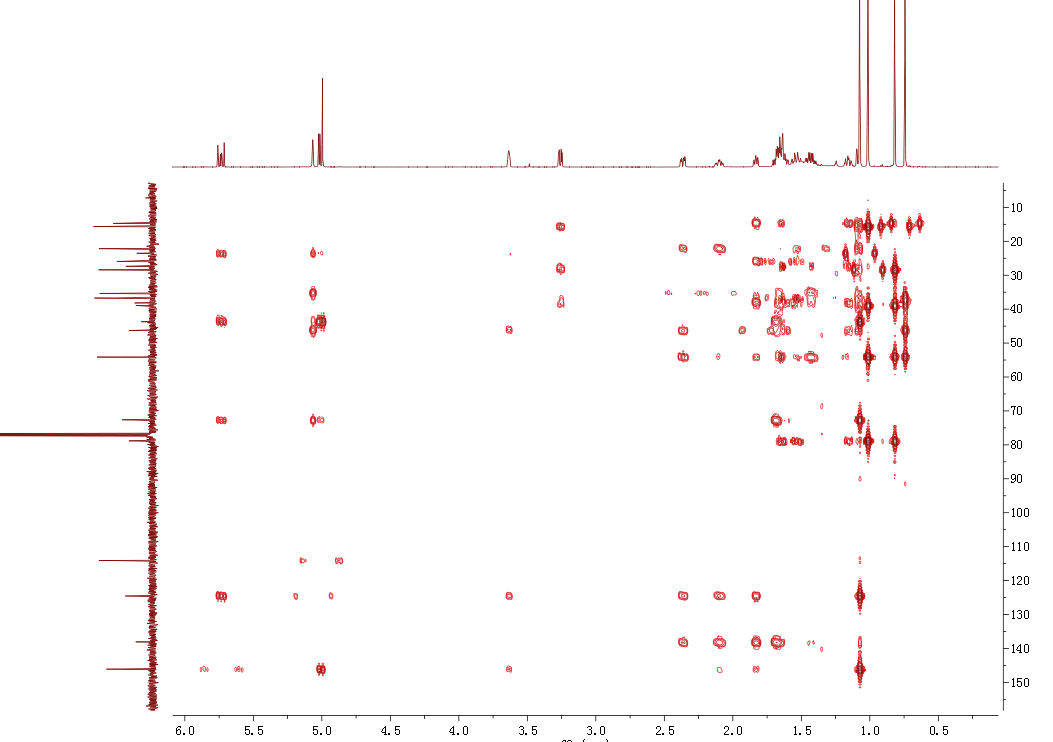


**Figure S5.** HMBC spectrum of compound **1** (CDCl_3_)


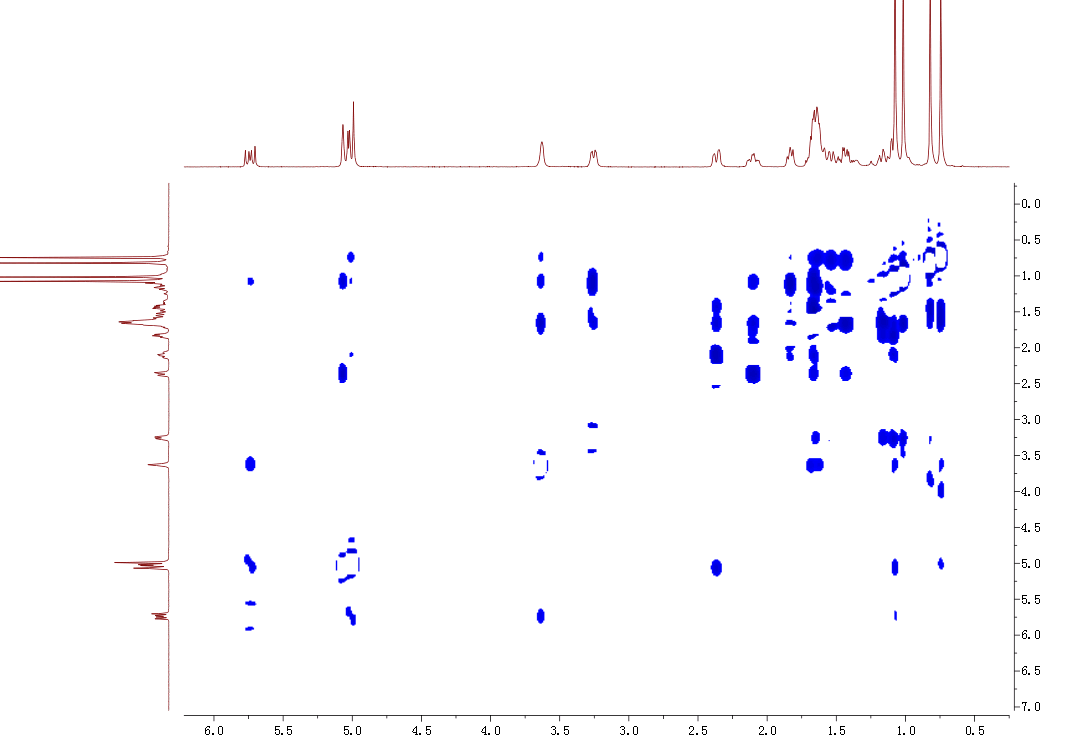


**Figure S6.** ROESY spectrum of compound **1** (CDCl_3_)


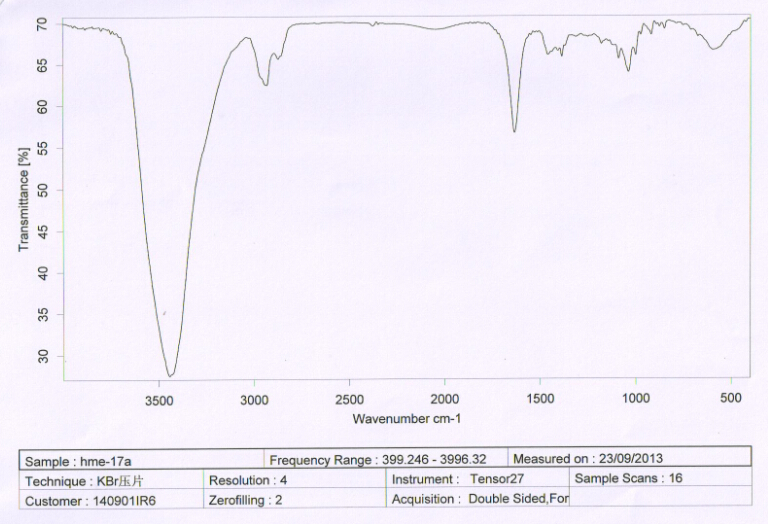


**Figure S7.** IR spectrum of compound **1** (KBr disks)


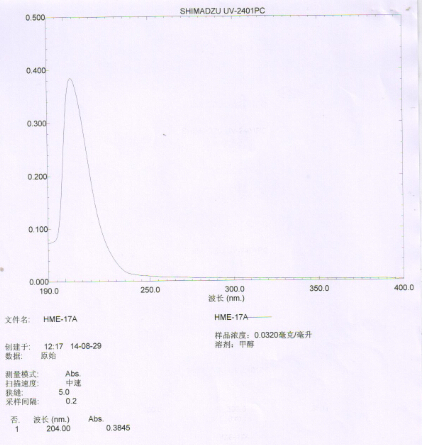


**Figure S8.** UV spectrum of compound **1** (MeOH)


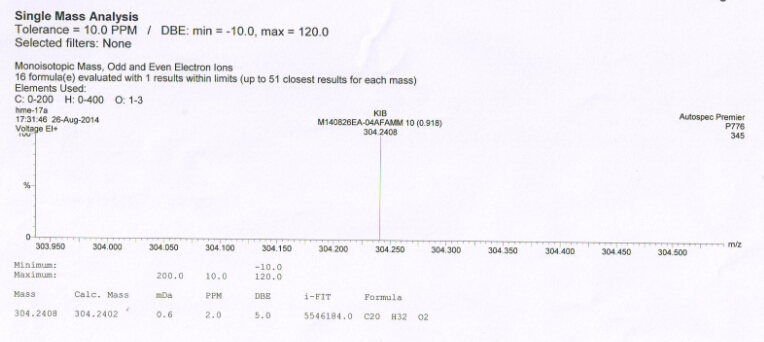


**Figure S9.** HREIMS of compound **1**

.


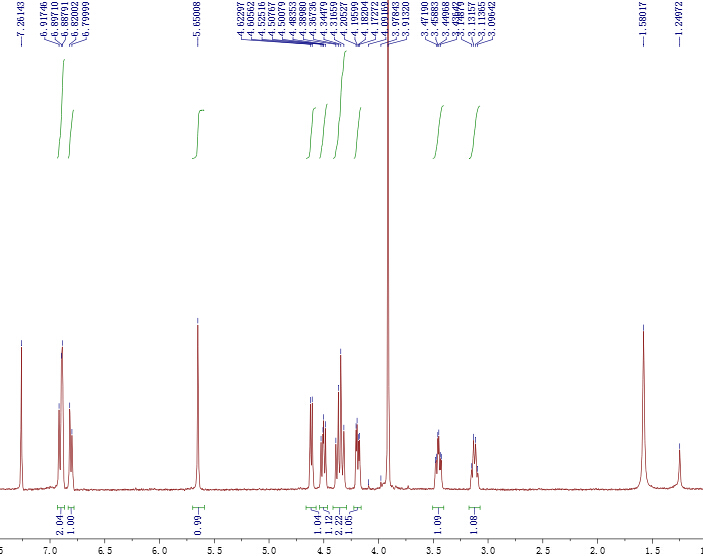


**Figure S10.** ^1^H NMR spectrum of compound **6** (CDCl_3_, 400 MHz)


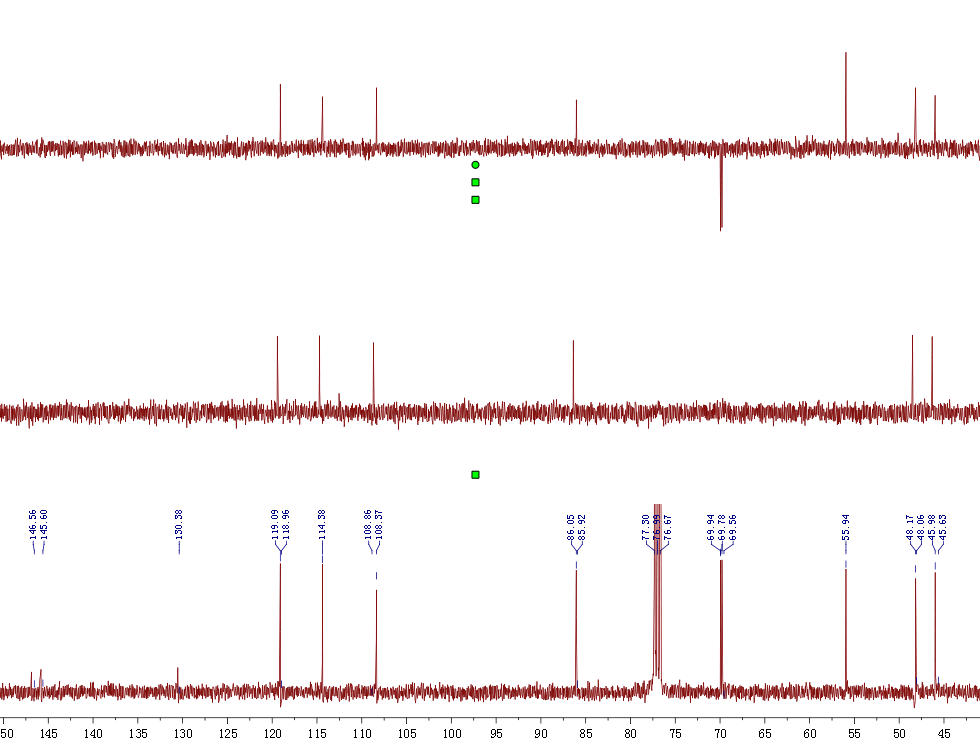


**Figure S11.** ^13^C NMR and DEPT spectrum of compound **6** (CDCl_3_, 100 MHz)


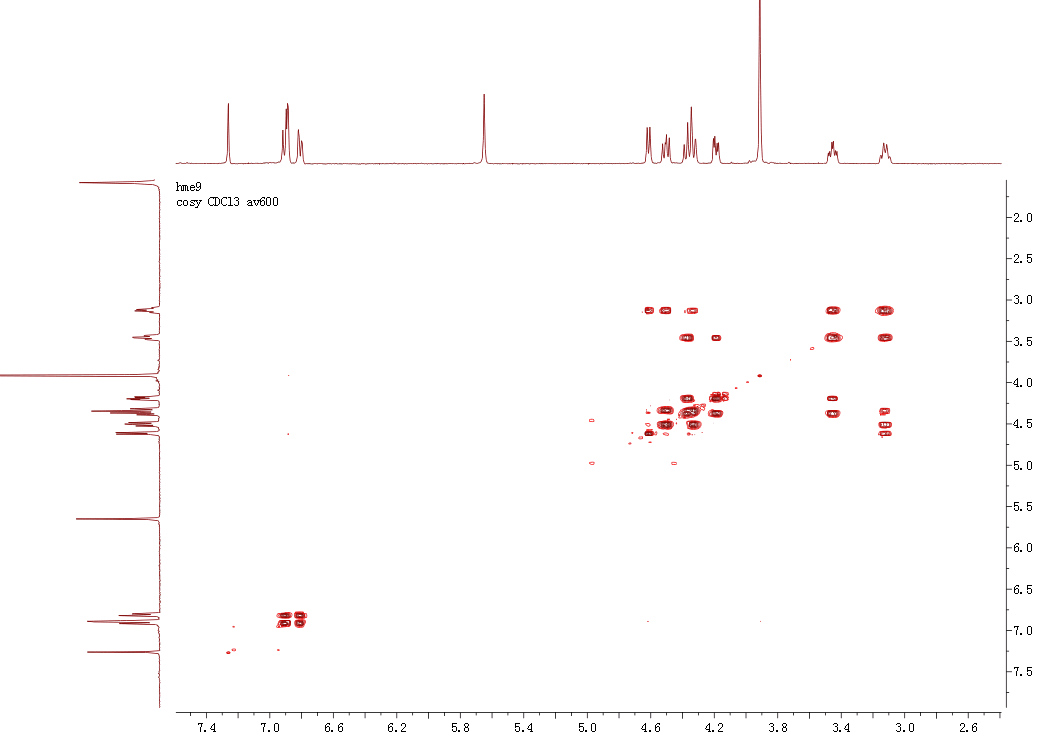


**Figure S12.** ^1^H-^1^H COSY spectrum of compound **6** (CDCl_3_)


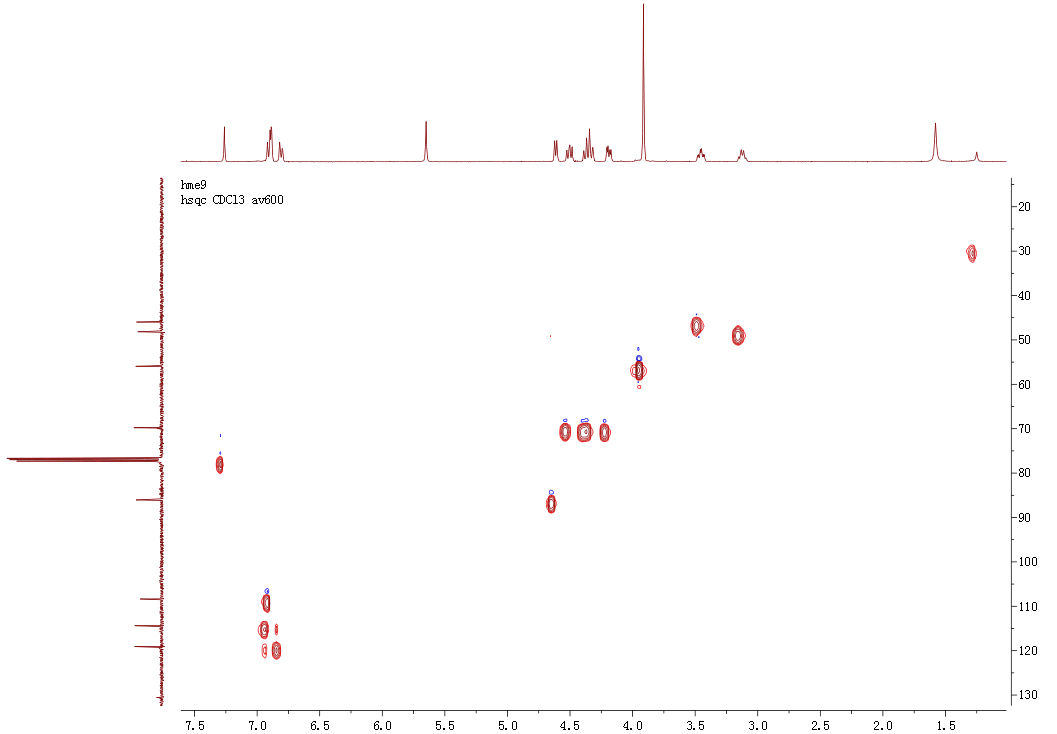


**Figure S13.** HSQC spectrum of compound **6** (CDCl_3_)


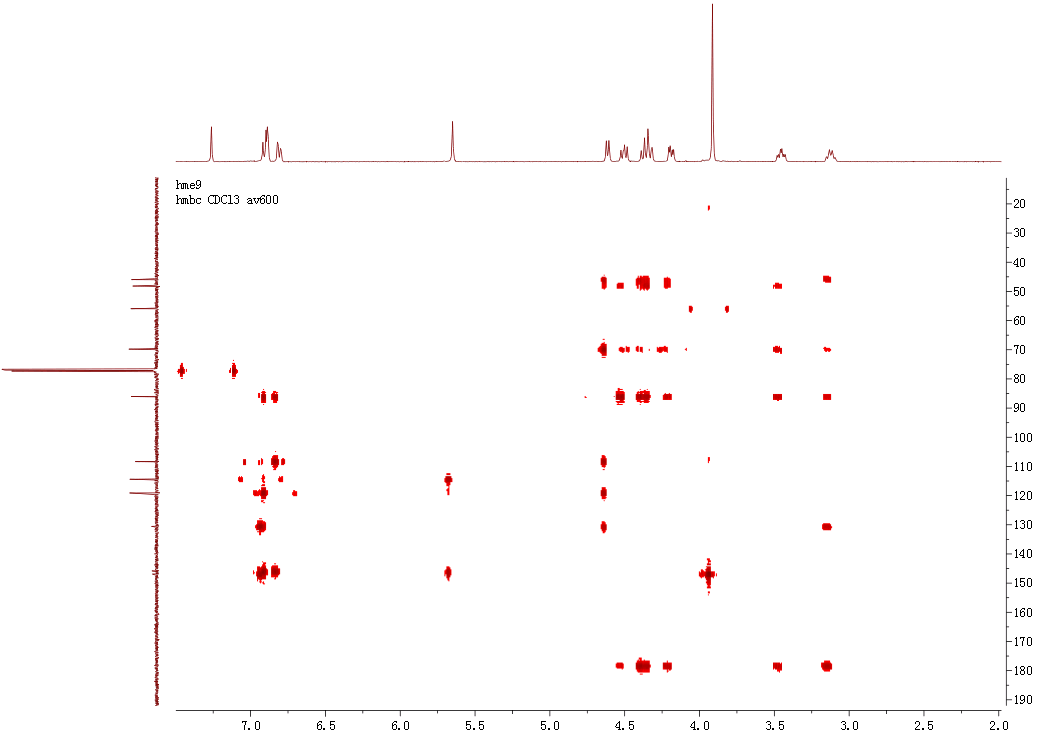


**Figure S14.** HMBC spectrum of compound **6** (CDCl_3_)


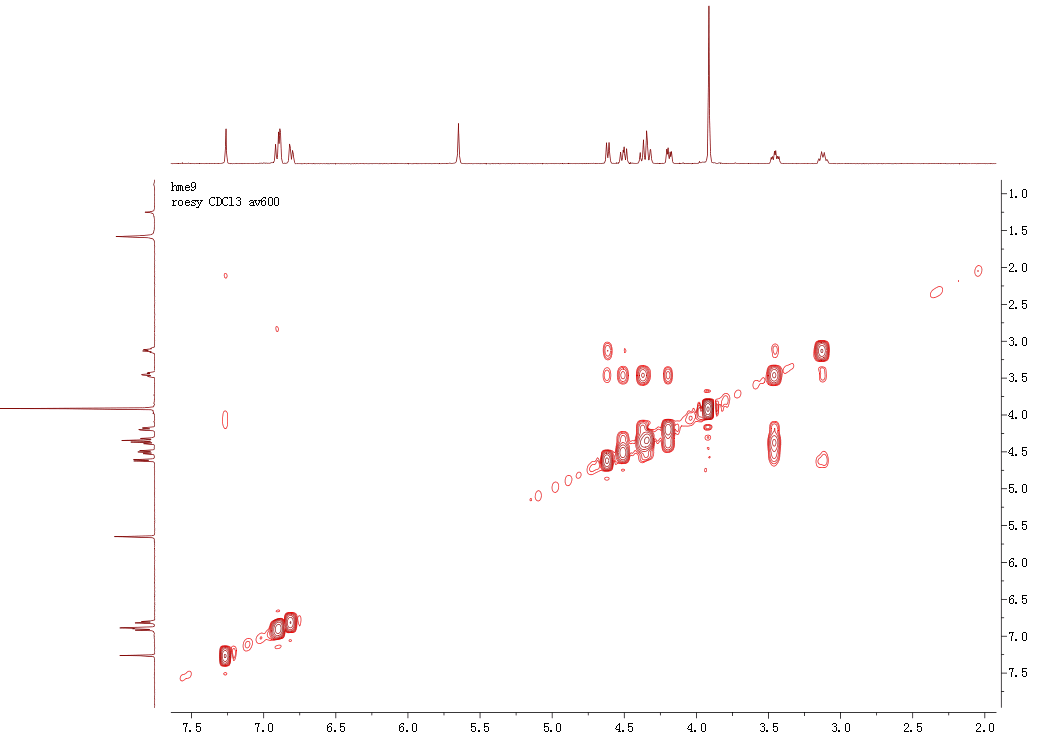


**Figure S15.** ROESY spectrum of compound **6** (CDCl_3_)


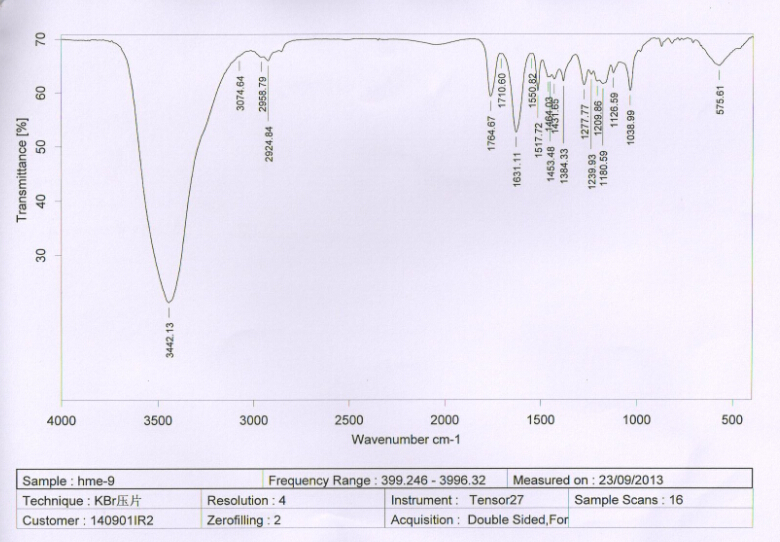


**Figure S16.** IR spectrum of compound **6** (KBr disks)


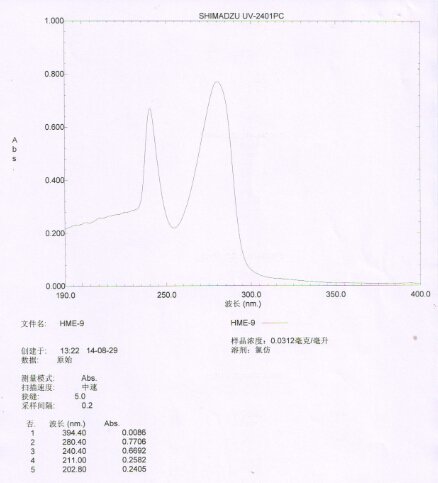


**Figure S17.** UV spectrum of compound **6** (MeOH)


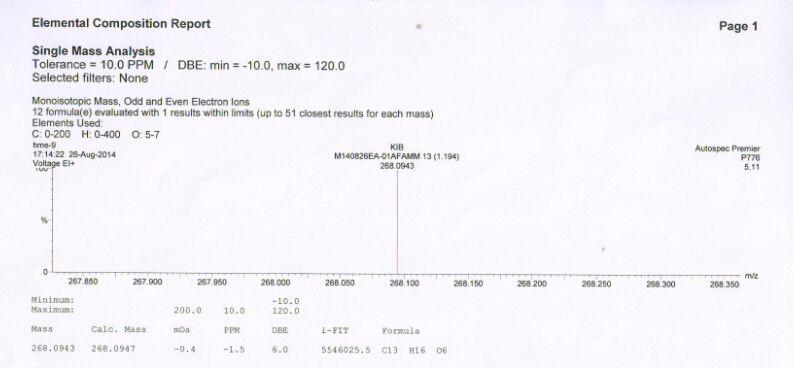


**Figure S18.** HREIMS of compound **6**
